# Effects of Medium and Temperature on Cellular Responses in the Superficial Zone of Hypo-Osmotically Challenged Articular Cartilage

**DOI:** 10.3390/jfb3030544

**Published:** 2012-08-09

**Authors:** Mari Huttu, Siru Turunen, Viktoria Sokolinski, Virpi Tiitu, Mikko Lammi, Rami K. Korhonen

**Affiliations:** 1 Department of Applied Physics, University of Eastern Finland, P.O. Box 1627, Kuopio FI-70211, Finland; Email: Mari.Huttu@uef.fi (M.H.); Siru.Turunen@uef.fi (S.T.); sokolins@hytti.uku.fi (V.S.); Rami.Korhonen@uef.fi (R.K.); 2 Institute of Biomedicine, University of Eastern Finland, P.O. Box 1627, Kuopio FI-70211, Finland; Email: Virpi.Tiitu@uef.fi; 3 SIB-Labs, University of Eastern Finland, P.O. Box 1627, Kuopio FI-70211, Finland; 4 Biocenter Kuopio, University of Eastern Finland, P.O. Box 1627, Kuopio FI-70211, Finland

**Keywords:** chondrocytes, medium, temperature, confocal microscopy, osmolarity

## Abstract

Osmotic loading of articular cartilage has been used to study cell-tissue interactions and mechanisms in chondrocyte volume regulation *in situ*. Since cell volume changes are likely to affect cell’s mechanotransduction, it is important to understand how environmental factors, such as composition of the immersion medium and temperature affect cell volume changes *in situ* in osmotically challenged articular cartilage. In this study, chondrocytes were imaged *in situ* with a confocal laser scanning microscope (CLSM) through cartilage surface before and 3 min and 120 min after a hypo-osmotic challenge. Samples were measured either in phosphate buffered saline (PBS, without glucose and Ca^2+^) or in Dulbecco’s modified Eagle’s medium (DMEM, with glucose and Ca^2+^), and at 21 °C or at 37 °C. In all groups, cell volumes increased shortly after the hypotonic challenge and then recovered back to the original volumes. At both observation time points, cell volume changes as a result of the osmotic challenge were similar in PBS and DMEM in both temperatures. Our results indicate that the initial chondrocyte swelling and volume recovery as a result of the hypo-osmotic challenge of cartilage are not dependent on commonly used immersion media or temperature.

## 1. Introduction

One of the earliest signs of osteoarthritis (OA) is swelling of articular cartilage [1,2,3]. OA leads to decreased osmolarity and increased chondrocyte volume [1,4], which may affect cartilage biosynthesis [1,5,6,7]. The extracellular osmolarity in healthy cartilage ranges approximately between 350 and 450 mOsm [7]. In OA, the osmotic environment of chondrocytes becomes hypotonic and the extracellular osmolarity can be reduced even down to ~270 mOsm [1,4]. Osmotic challenges have been typically applied for chondrocytes *in situ* to mimic the altered physico-chemical environment of chondrocytes present in OA [1,6,8]. Previous studies have been conducted either in phosphate-buffered saline (PBS) or Dulbecco’s modified Eagle’s medium (DMEM), and either in physiological or at room temperature [1,4,6,8,9,10]. It has not been shown whether these different environmental factors affect chondrocyte volume changes.

It is known that chondrocyte volumes increase rapidly (some minutes) in response to a hypotonic challenge [6,8,10,11,12]. It has been shown in many previous studies that, after the initial cell swelling, the full cell volume recovery occurs within 20 min after the osmotic challenge [4,6,13]. On the other hand, some have measured cells *in situ* for hours and did not see any difference in cell volumes between 20 min and 120 min, even though cell volume recovery was dependent on the tissue integrity [12]. 

Two major energy sources of mammalian cells are glutamine and glucose. In anaerobic glycolysis, the yield of ATP is 2 mol per mole of glucose, while in aerobic conditions, the yield of ATP is 36 mol per mole of glucose [14,15]. Since articular cartilage is an avascular tissue, the main energy source for chondrocytes is glycolysis [16]. 

Ion transporters provide the most rapid and efficient cell volume regulatory mechanisms, which following cellular swelling, activate to restore cell volume by transporting ions out of the cell [17]. This cell volume regulation is expected to be disturbed by the lack of energy for cells. Calcium (Ca^2+^) is also thought to be involved in cell volume regulation [18]. Thus, we hypothesize that, following the initial cell swelling after the hypotonic challenge, cells recover better back to the original volume in DMEM than in PBS, because only DMEM contains glucose and Ca^2+^ (Table 1). 

**Table 1:** Main components in the isotonic Dulbecco’s modified Eagle’s medium (DMEM) and phosphate-buffered saline (PBS) used in this study. Concentrations (mg/L) of inorganic salts have also been indicated.

Component	DMEM 290 mOsm	PBS 290 mOsm
Amino Acids	×	–
Vitamins	×	–
D-glucose (Dextrose)	×	–
Sodium Chloride (NaCl)	6,400 mg/L	8,000 mg/L
Potassium Chloride (KCl)	400 mg/L	200 mg/L
Sodium Phosphate monobasic (NaH_2_PO_4_)	141 mg/L	–
Sodium Phosphate dibasic (Na_2_HPO_4_)	–	1,440 mg/L
Potassium Phosphate monobasic (KH_2_PO_4_)	–	240 mg/L
Calcium Chloride (CaCl_2_) anhyd.	264 mg/L	–
Ferric Nitrate (Fe(NO_3_)_3_''9H_2_O)	0.1 mg/L	–
Magnesium Sulfate (MgSO_4_) anhyd.	200 mg/L	–
Sodium Bicarbonate (NaHCO_3_)	3,700 mg/L	–

Many previous experiments of chondrocyte volume changes have been conducted at room temperature (~21 °C) [4,6,8]. Cell volume changes in osmotically challenged cartilage may be altered by the functions of the ‘osmolyte channel’ of cells that are thought to be relatively sensitive to temperature [19]. Microtubules and microfilaments are important constituents of the cytoskeleton of cells, and they are known to be temperature-sensitive. Lower temperatures can affect the assembly of the cellular cytoskeleton and may change the stiffness of the cell membrane-linked microfilament network. Changes in the microfilament network can potentially change the extent how much the cortical actin can resist the changes in cell shape. On the other hand, it has been suggested that in higher temperatures microtubules are more instable, which could alter cell stiffness and volume [20]. In fact, it was shown earlier that the stiffness of human mesenchymal stem cells is reduced in higher temperatures. Thus, we hypothesize that the cell volume initially (within minutes) increases more in hypo-osmotically challenged cartilage at physiological temperature (37 °C) than at room temperature (21 °C). It has not been shown directly whether environmental factors, such as composition of the immersion medium and temperature affect cell volume changes *in situ* in a relatively short (hours) osmotic loading experiment of articular cartilage. The aim of this study was to compare the influence of two different media (PBS and DMEM) on cell volume changes in the superficial zone of hypo-osmotically challenged bovine articular cartilage in two different temperatures (21 °C and 37 °C). We primarily hypothesized that cell volumes would first increase but not recover back to the original volume in PBS [8,12], while in DMEM cells would recover back to the original, unchallenged volume [6]. Secondary hypothesis was that and the initial cell volume increase following the hypo-osmotic challenge of cartilage would be greater at 37 °C than at 21 °C. This study provides a controlled characterization of cell volume changes in osmotically challenged articular cartilage when the cell environment is manipulated, and points out the importance of the cell environment on the results and conclusions of *in vitro*/*in situ* laboratory studies when relating the findings to real life. 

## 2. Experimental Section

### 2.1. Sample Preparation

**Figure 1:**
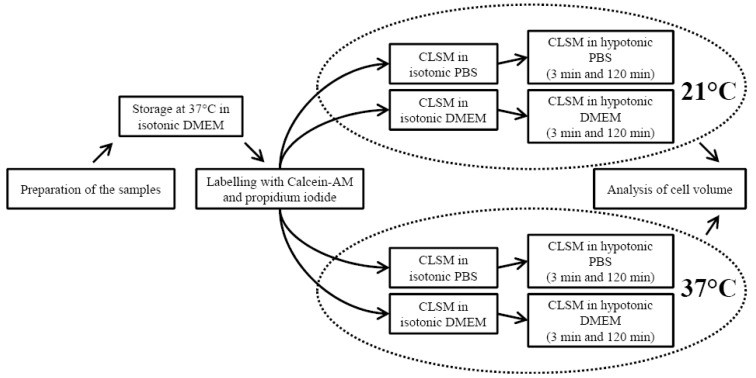
Diagram of the study protocol.

**Figure 2:**
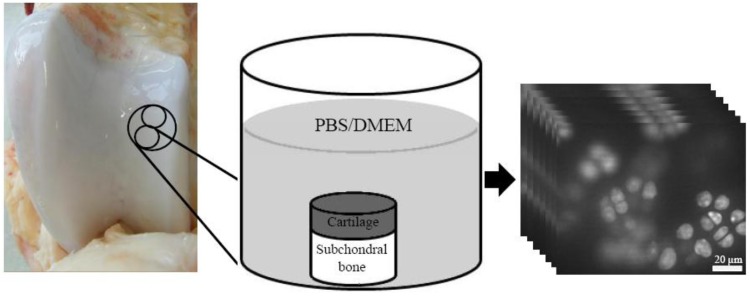
Preparation of the samples for confocal laser scanning microscope (CLSM). Two adjacent samples with subchondral bone were freed from the lateral patellar groove. The samples were incubated for 24 or 48 hours until the hypo-osmotic challenge and CLSM imaging took place. A representative image stack is indicated on the right.

For both temperature groups (21 °C and 37 °C), seven bovine knee joints (age 16–23 months) were obtained from the local slaughterhouse (Figure 1 and Figure 2). Articular cartilage samples with subchondral bone were released from the lateral patellar groove (LPG) of the femur using a drill bit (diameter = 21 mm) (Figure 2). Two smaller cylindrical plugs with subchondral bone were further prepared using a metallic punch (diameter = 10 mm) (Figure 2). In both temperature groups, the other cylindrical plug was tested in PBS and the adjacent one in DMEM (1 g/L D-glucose, L-glutamine- and phenol red-free, Invitrogen, Paisley, UK) (Figure 1). Isotonic (osmolarity ~290 mOsm) and hypotonic (osmolarity ~170 mOsm) PBS and DMEM were prepared simultaneously before any experiment took place. Different PBS solutions were prepared by using different amounts of NaCl. Original osmolarity of DMEM was ~290 mOsm, and the hypotonic DMEM was prepared by increasing distilled water in the solution. The same methods for preparing PBS and DMEM have been used in several previous studies of chondrocyte responses to osmotic challenges [1,5,6,8,10,12,21], though changing osmolarity by using different amounts of NaCl introduces a potentially confounding factor that is not present when an inert osmolyte is employed.

As stated in the introduction, PBS does not provide supplements for glucose or calcium, and DMEM contains physiological levels of glucose and calcium (Table 1). In each group, chondrocytes were studied at the center of the cylindrical plugs through the cartilage surface, similarly as in earlier studies [8,10,12,22]. 

**Figure 3:**
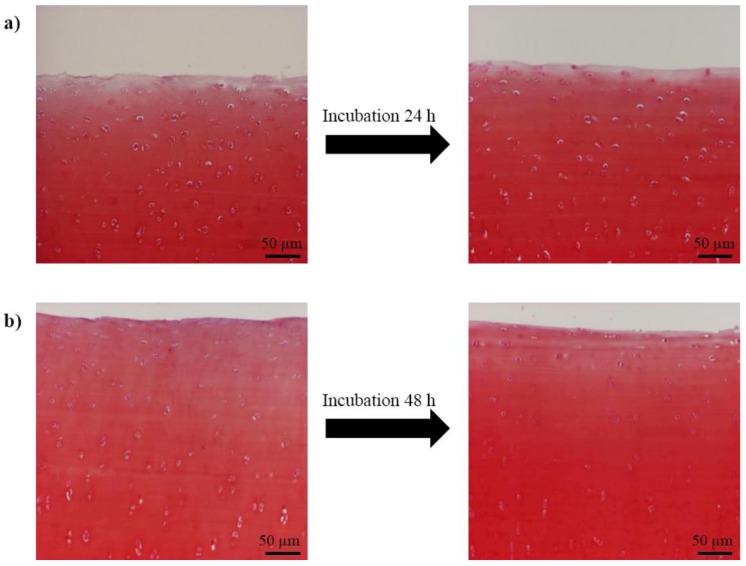
Qualitative analysis of proteoglycan (PG) content of articular cartilage for the samples processed for microscopy instantly after the sample preparation (left) and for the samples incubated first 24 h (**a**) and 48 h (**b**) and then processed for microscopy. Images of 3 µm thick histological Safranin-O stained sections [23,24,25] were obtained with a standard light microscope.

Prior to imaging with confocal laser scanning microscope (CLSM), the samples were kept in DMEM supplemented with 100 U/mL of penicillin, 100 µg/mL of streptomycin (EuroClone S.p.A, Pavia, Italy) and 2.50 µg/mL Fungizone (amphotericin-B) (Invitrogen, Paisley, UK) in the incubator (37 °C) for 24 or 48 hours. Fungizone was necessary for mould prevention during incubation. Paired samples, *i.e.*, the samples from the same joint were always kept equal time in the incubator. Qualitative analysis indicated that the incubation did not change cartilage composition and integrity, especially the proteoglycan leakage through the cartilage surface was negligible (Figure 3) [23,24,25]. After incubation, the samples were stained for 30 min with both calcein-AM (5 µM, Invitrogen, Oregon, USA) and propidium iodide (60 µM, Sigma, New York, NY, USA) for live and dead cells, respectively. Calcein-AM and propidium iodide have been used together in many studies to assess cell volume and cell death, respectively [1,4,9], and their concentrations were based on earlier studies [1,4,5,6,8,10,12,13]. The staining solutions (and subsequently the solutions used in the experiments) were made to isotonic PBS (osmolarity ~290 mOsm) or DMEM (osmolarity ~290 mOsm) without additives, and the samples were stained either at room temperature or at 37 °C (Figure 1).

### 2.2. Confocal Laser Scanning Microscopy

A Nikon Eclipse TE-300 microscope (Nikon Corporation, Japan) with ultraVIEW confocal laser scanner (PerkinElmer Life Sciences, Boston, MA, USA) was used for cell imaging [12]. Stacks of images were obtained with a 0.3 µm vertical z-axis spacing using an objective with 60× magnification. The vertical resolution was determined by imaging polystyrene fluorescent microspheres (Polyscience Europe GmbH, Eppelheim, Germany) with the diameter of 7.3 µm (volume 203.7 µm^3^). The pixel size in x-y plane was 0.2 µm × 0.2 µm (672 × 512 pixel images). Laser excitation wavelengths for live and dead cells were 488/10 nm and 568/10 nm, respectively, and fluorescences were measured with laser emission wavelengths of 525/50 nm and 607/45 nm. Laser power was adjusted to provide optimum image quality with minimum dye bleaching. 

Chondrocytes in each group were first imaged in isotonic medium (~290 mOsm) (Figure 1). Then, the medium concentration was changed to hypotonic (~170 mOsm) by a rapid aspiration in about 30 s. Image stacks were further captured at two different time points, *i.e.*, at short-term (3 min) and at steady-state (120 min) time point [8,12]. The short-term time point was chosen to study the differences in the initial cell swelling between the temperature groups, and the long-term time point was chosen to clarify whether the cell volume would recover back to the original volume in PBS after the initial volume increase. Based on earlier studies, this long-term time point should be enough for the cells to reach the steady state [4,6,13], and there are earlier studies that have measured cell volumes or cell viability for hours [8,12,21,26]. On the other hand, excessively long tests could cause, e.g., compartmentalization of the dye and uncertainties in the cell volume calculation. Images were captured up to ~60 µm depth through the cartilage surface. There was a loss of fluorescent signal at greater depths. 

### 2.3. Data Analysis

Image stacks were analyzed with ImageJ (National Institute of Health, Bethesda, MD, USA). Five to ten cells from each image stack were chosen randomly for the analysis; however, location of the analyzed cells was at about in the middle of each image stack. Before choosing the cells for the analysis, propidium iodide stained cells were identified to check cell viabilities. Dead and badly photobleached cells were rejected from further analysis, and only cells with bright green fluorescence were analyzed. The Visualization Toolkit 5.2.0 (Kitware Inc., New York, NY, USA) was used to reconstruct 3D-images of the cells, and a Python code was used to calculate cell volumes at each time point [8,10,12,22,27]. Threshold of 40% of the maximum fluorescence intensity of each cell individually, the same as used earlier [4,6,8,10,12], was used for cell volume calculations.

### 2.4. Statistical Analysis

The results are presented as mean ± s.d. The number of samples and cells has been indicated as *n* and *N*, respectively. Since the samples immersed in either PBS or DMEM were from the same joint, and were thus related (or paired), but at the same time the analyzed cells were different between the groups, statistical comparisons in the normalized cell volumes between the medium groups (PBS *vs.* DMEM) were performed using a mixed linear model [12,28,29,30]. The same model was used in the comparisons of the normalized cell volumes between different time points. In this model the time of measurement, type of sample and interactions between them were set as fixed variables, and the sample number was set as the random variable. Mann-Whitney U test was used to test significant differences in normalized cell volumes between the temperature groups (21 °C *vs*. 37 °C), since the samples immersed in different temperatures were from different joints, and thus, they were independent. SPSS 14.0 was used for statistical comparisons (SPSS Inc., Chicago, IL, USA). The results were considered significantly different when the *p* value was less than 0.05.

## 3. Results

At 21 °C and 37 °C, average absolute chondrocyte volumes in the isotonic PBS were 436 ± 44 µm^3^ and 421 ± 76 µm^3^, respectively. Corresponding cell volumes in the isotonic DMEM were 446 ± 48 µm^3^ and 499 ± 50 µm^3^, respectively. Average absolute chondrocyte volumes at 37 °C were significantly greater (*p* < 0.05) in the isotonic DMEM than in the isotonic PBS. 

**Figure 4:**
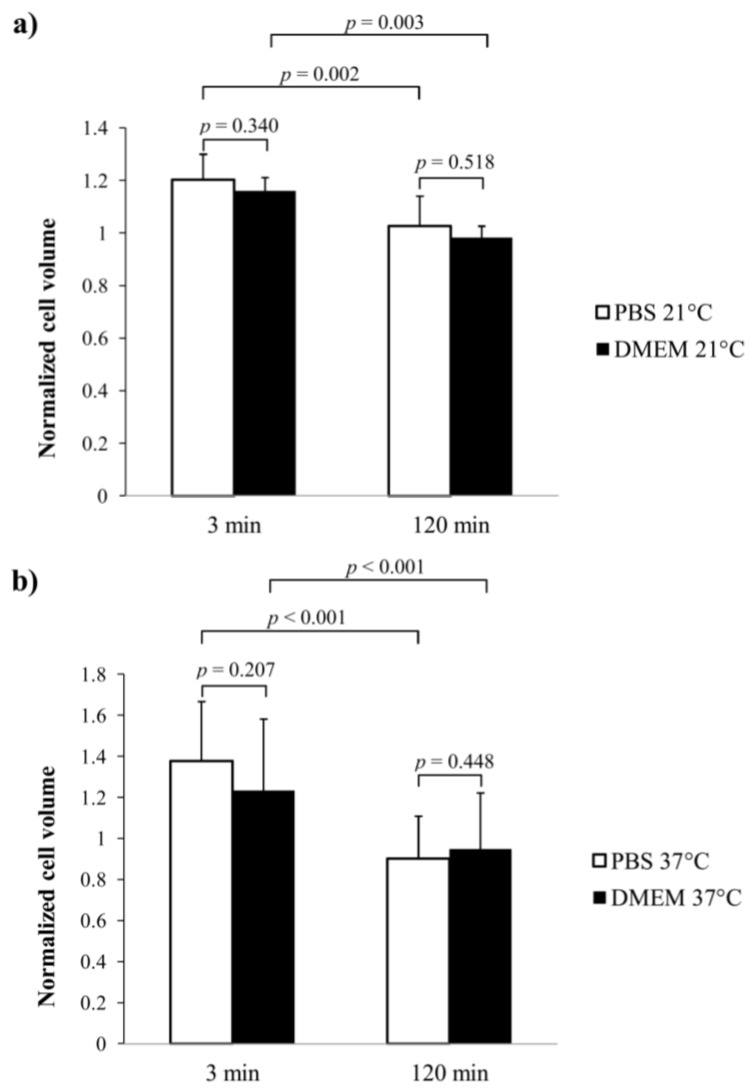
Cell volume change (normalized cell volume) in the samples immersed in two different media (PBS and DMEM) and at temperatures of 21 °C (**a**) and 37 °C; (**b**) after changing the medium concentration from isotonic to hypotonic. Cells were imaged and cell volumes analyzed before the osmotic challenge and at two different time points (3 min and 120 min) after the osmotic shock. The cell volumes at 3 and 120 min time points were normalized with those recorded before the osmotic challenge.

In both temperatures (21 °C and 37 °C) and media (PBS and DMEM), chondrocyte volumes (*n* = 7/group, *N* = 5–10/sample) were significantly greater (*p* < 0.05) three min after the medium was changed from isotonic to hypotonic, as compared to the original, unchallenged cell volumes. After the initial cell swelling, chondrocyte volumes decreased significantly (3 min *vs.* 120 min, *n* = 7/group, *N* = 5–10/sample, *p* < 0.05) and reached original volumes within the 120 min measurement period (0 min *vs.* 120 min, *n* = 7/group, *N* = 5–10/sample, *p* > 0.05) (Figure 4). 

In both observation time points, *i.e.*, 3 and 120 min after the change of the medium from isotonic to hypotonic, there were no statistically significant differences (*n* = 7/group, *N* = 5–10/sample, *p* > 0.05) in normalized cell volumes between the samples immersed in PBS or DMEM (Figure 4). This result was the same at 21 °C and 37 °C. Further, in both observation time points, normalized cell volumes were not significantly different (*n* = 7/group, *N* = 5–10/sample, *p* > 0.05) for the samples measured at 21 °C or 37 °C. This result was the same for the samples immersed in PBS and DMEM.

## 4. Discussion

We studied the effect of two commonly used media on short- (3 min) and long- (120 min) term cell volume changes *in situ* following hypo-osmotic loading of bovine articular cartilage in two different temperatures. Chondrocytes were imaged in isotonic and hypotonic PBS or DMEM at room temperature (21 °C) or at physiological temperature (37 °C) through cartilage surfaces using CLSM. In all groups, cell volumes increased shortly after the hypo-osmotic challenge and then recovered back to the original volumes. Interestingly, cell volume changes at both observation time points were not different for the samples immersed in PBS or DMEM, and for the samples measured at 21 °C or at 37 °C.

The absolute volumes of chondrocytes in the isotonic media were consistent with earlier studies [4,5,8]. The initial cell volume increase (~20%) in hypo-osmotically challenged articular cartilage, as also detected earlier [5,6,21], was likely due to the changed osmotic pressure gradient, *i.e.* the osmotic pressure difference in and out of cells and subsequent fluid flow into cells. As a result of active cell volume regulation processes, cells recovered back to the original volume during the observation period. This is also consistent with several earlier studies [6,19,21,31].

Environmental stresses, such as glucose deprivation, have been noticed to rapidly shut down translation initiation and actin polarization [32,33]. Since actin network surrounds the chondrocytes and is directly in contact with the extracellular matrix (ECM) components via integrins, it can be assumed that the maintenance of this link might be affected when cells are cultured in glucose depleted PBS (Table 1). This is one reason why in hypo-osmotic loading experiments, cell volume recovery after the initial volume increase may be inhibited by the use of PBS [15]. However, our results suggest otherwise. Even though articular cartilage is an avascular tissue and the main energy source for chondrocytes is glucose, which in addition to Ca^2+^ is not present in PBS, the metabolism of chondrocytes is relatively slow. Thus, it may be that two hours is still such a short measurement period that cells receive enough nutrients even without the use of DMEM. This is supported by the results from an earlier study which indicated that cells exposed to low glucose levels were able to use more affordable metabolic pathways, and thus lower rate of nourishment did not affect the ECM synthesis rates [34]. Most likely cell volume changes in the samples immersed in PBS and DMEM would differ in longer experiments. However, this kind of short experiments from minutes to hours as applied here are commonly used [5,6,9,10,31], since cell volume changes after hypo-osmotic loading are rapid [6,10]. It should also be remembered here that our goal was not to study specifically the effect of glucose or Ca^2+^ on cell volume changes, but the effect of two commonly used media on cell volume changes, one of which is lacking glucose and Ca^2+^ (Table 1). Thus, our results suggest that it is safe to use PBS in osmotic loading experiments of cells *in situ*, at least if the measurement lasts only minutes or a few hours.

In contrast to our hypothesis, initial cell swelling in hypo-osmotically challenged cartilage was not dependent on temperature. This suggests that reduction of the cell membrane stiffness, which has earlier been suggested to be temperature-dependent [20], was minor or negligible and did not have a role in modulating cell volumes. On the other hand, cell volume regulation after the initial cell swelling in hypo-osmotically challenged cartilage has earlier been suggested to be faster at 37 °C than at 21 °C [6,31,35]. In this study we did not want to concentrate on the recovery rate, since then images should have been captured at least in one minute intervals. That would have caused dye bleaching and risk the most important long-term time point. That would have also forced us to reduce scanning resolution, which could have eventually led to the loss of data and uncertainties/errors in cell volume calculation. However, these limitations should be overcome for instance by the use of dual photon microscope [1] and more cell volume recovery times should be studied in the future. 

In previous studies of chondrocyte response to osmotic challenge, the first observation time point has typically been 1.5 min or more [5,6,8,10,12]. Earlier studies have been conducted for isolated cells, cells in explant tissues or cells in fully intact cartilage tissue. The initial cell swelling might be faster for isolated cells than those in fully intact tissue. Thus, we chose a lightly longer short-term time point (3 min) than used earlier in imaging of isolated cells. Due to the choice of only one short-term time point, it is not certain whether chondrocytes are still expanding, in their peak volume or even already recovering at that observation time point. This is a limitation of our study which should be overcome in the future by imaging cells in several observation time points. However, as discussed above it was not possible in the present study.

Samples for the different medium groups were obtained from the same joints giving higher power to the statistical analysis. In these statistical tests, ~50 or more cells were analyzed for each group at each time point. For technical reasons, the samples for different temperature groups (21 °C and 37 °C) were, however, obtained from separate knee joints and the number of samples was relatively low. To increase the power of the statistical analyses, significant differences were also studied in combined groups; medium groups with both temperature groups together and temperature groups with both medium groups together. Even in these analyses, statistical significances in the normalized cell volumes were not observed. Statistical significances between in the normalized cell volumes between the groups could reach even the value of 0.518. To substantiate our results, paired samples should be prepared and analyzed in the future to study the effect of temperature on cellular responses following hypo-osmotic loading. 

Measurements were conducted through cartilage surfaces. This kind of samples have been used also in earlier experiments [8,10]. Thus, cutting of the samples was not needed before imaging, as has been done in many earlier studies [1,4,5,6]. This minimized the possible problems occurring in the vicinity of the cut edge (e.g. loosening of the collagen fibril tension) that might ultimately affect cell volume changes. With our technique, we were only able to measure cells up to ~60 µm depth through the cartilage surface, and the analyzed cells were at about 30–40 µm deep in the tissue (less than 5% of cartilage thickness). Thus, we measured cells only in the superficial zone [36,37,38]. In order to be able to measure cells in the deeper zones, the only current option would be to cut the samples and measure cells through the cut surface [1,4,5,6]. However, since cutting of the samples may affect the 3D-structure of articular cartilage and it has also been shown that chondrocyte responses as measured through the cut edge and through the cartilage surface are different [5,8,12], we measured only intact cartilage in this study.

Cell-tissue interactions are likely to modulate hypotonic-induced cell swelling and volume recovery [6,7]. Those interactions could be different in DMEM and PBS. This could be clarified by studying isolated cells in PBS and DMEM and by comparing the results with those obtained for *in situ* cells.

## 5. Conclusions

Our results suggest that the initial cell volume increase and subsequent volume recovery in hypo-osmotically challenged articular cartilage are not dependent on the composition of commonly used immersion media or temperature. Overall, we report important methodological and quality control findings and emphasize the importance of the cell environment when relating *in vitro*/*in situ* findings to real life.
